# Impact of Thermomechanical Fiber Pre-Treatment Using Twin-Screw Extrusion on the Production and Properties of Renewable Binderless Coriander Fiberboards

**DOI:** 10.3390/ijms18071539

**Published:** 2017-07-17

**Authors:** Evelien Uitterhaegen, Laurent Labonne, Othmane Merah, Thierry Talou, Stéphane Ballas, Thierry Véronèse, Philippe Evon

**Affiliations:** 1 Laboratoire de Chimie Agro-industrielle (LCA), Université de Toulouse, Institut National de la Recherche Agronomique (INRA), Institut National Polytechnique de Toulouse (INPT), 31030 Toulouse CEDEX 4, France; Evelien.Uitterhaegen@ensiacet.fr (E.U.); Laurent.Labonne@ensiacet.fr (L.L.); Othmane.Merah@ensiacet.fr (O.M.); Thierry.Talou@ensiacet.fr (T.T.); 2 Ovalie Innovation, 2 Rue Marguerite Duras, 32000 Auch, France; stephane.ballas@ovalie-innovation.com (S.B.); thierry.veronese@ovalie-innovation.com (T.V.)

**Keywords:** binderless fiberboards, fiber refining, thermopressing, *Coriandrum sativum* L., press cake, proteins

## Abstract

The aim of this study consisted of manufacturing renewable binderless fiberboards from coriander straw and a deoiled coriander press cake, thus at the same time ensuring the valorization of crop residues and process by-products. The press cake acted as a natural binder inside the boards owing to the thermoplastic behavior of its protein fraction during thermopressing. The influence of different fiber-refining methods was evaluated and it was shown that a twin-screw extrusion treatment effectively improved fiber morphology and resulted in fiberboards with enhanced performance as compared to a conventional grinding process. The best fiberboard was produced with extrusion-refined straw using a 0.4 liquid/solid (L/S) ratio and with 40% press cake addition. The water sensitivity of the boards was effectively reduced by 63% through the addition of an extrusion raw material premixing operation and thermal treatment of the panels at 200 °C, resulting in materials with good performance showing a flexural strength of 29 MPa and a thickness swelling of 24%. Produced without the use of any chemical adhesives, these fiberboards could thus present viable, sustainable alternatives for current commercial wood-based materials such as oriented strand board, particleboard and medium-density fiberboard, with high cost-effectiveness.

## 1. Introduction

Recent years have been marked by prominent environmental concerns and regulations, resulting from the continuously growing population and the steady depletion of both fossil and world forest resources. Therefore, the implementation of sustainable resources and processing has become of increasing importance in the materials industry, while current research is focusing on alternative sources of lignocellulosic fibers that could show a competitive nature with current commercial materials. In view of this, crop residues and lignocellulosic by-products from the processing of agricultural resources could provide a critical solution, as they exhibit low cost and high availability and do not lead to competition with the food industry for land use. Besides this, the use of often toxic, hazardous resins such as urea formaldehyde, has led to environmental, as well as health and safety concerns. Recently, awareness has been raised and regulations have been set regarding indoor air quality, as the interior use of panels glued with formaldehyde resins may result in the formation of toxic formaldehyde emissions [1]. Furthermore, synthetic adhesives are relatively expensive and often represent over 30% of the total production cost for particleboard or fiberboard [2].

Therefore, research interest has shifted towards the use of natural binders and further to the production of binderless boards, thus avoiding the use of any chemical additives [3]. Cohesive binderless boards with good performance have been successfully manufactured through the thermopressing of lignocellulosic fiber material including wheat straw [4], *Miscanthus sinensis* [5], sugarcane bagasse [6], kenaf core [7,8], oil palm trunk [9] and banana bunch [10]. Here, the self-bonding capacity of the material is mainly attributed to the presence of lignins and, to a lesser degree, hemicelluloses. Lignins have been shown to have the capacity to act as a natural binder through their plasticization, which occurs when the glass transition temperature is surpassed under the high pressure and temperature conditions applied during thermopressing [11]. Then, lignins exhibit a matrix effect inside the manufactured board, embedding the cellulose fibers and resulting in cohesive, self-bonded panels [12,13,14]. Furthermore, it is believed that lignins and hemicelluloses undergo a certain degree of degradation under thermopressing, resulting in monomer blocks that induce cross-linking upon further condensation reactions and thus contribute to self-bonding [12,13,15,16]. Besides this, several research studies have demonstrated the bonding capacity of plant biopolymers such as starch and proteins owing to their thermoplastic behavior [17,18,19,20,21]. This has resulted in the recent use of press cake, a by-product resulting from the mechanical pressing of oilseeds, for the production of self-bonded boards [19,20,22,23,24,25].

With a view to the manufacture of binderless boards with improved mechanical properties and water resistance, pre-treatments involving fiber refining are often included in the production process. These fiber pre-treatments could consist of a grinding step, which presents a relatively simple operation to effectively reduce the fiber particle size, leading to an increased fiber surface area and accessibility to the inner cell wall components, in turn resulting in better molding and boards with improved mechanical performance [26,27]. Most commonly, however, fiber refining involves a steam treatment, which is often referred to as steam explosion, and is carried out in a thermomechanical refiner or digester. Many studies have shown the effectiveness of a steam pre-treatment for increasing board performance, in particular dimensional stability [5,8,10,28,29]. The process disrupts the fiber structure, liberates lignins from the inner cell wall to the fiber surface, hydrolyzes hemicelluloses, and to a lesser degree, lignins [3,30]. While the increased accessibility of lignins and the formation of reactive monomer compounds contribute to self-bonding during thermopressing, the reduced hemicellulose content, which shows strong hygroscopicity, results in improved water resistance [5,28]. Further, defibration separates the cellulose fibers, leading to an increase of the surface area and an improvement of the fiber morphology, in particular the aspect ratio, which represents the ratio of fiber length to width [8,29]. 

Coriander (*Coriandrum sativum* L.) is an annual herb belonging to the Apiaceae family and showing a global production of around 500,000 tons/year, which further results in about 500,000 tons/year of readily available coriander straw [31,32]. Coriander fruits are marked by the presence of both an essential oil fraction, typically representing less than 1% of the dry fruit weight, and a vegetable oil fraction of 20–28% of the dry fruit weight [33]. The vegetable oil has recently gained interest from the food, cosmetic, and chemical industries, owing to the presence of petroselinic acid as the major fatty acid (73% of all fatty acids) [34]. Petroselinic acid presents an uncommon positional isomer of oleic acid. It exhibits anti-inflammatory and anti-aging properties, and has been applied for the production of sophorolipid compounds [35,36,37]. Coriander vegetable oil has recently been approved as a novel food ingredient (NFI) and can be efficiently obtained from the fruits in high quality through mechanical pressing using a twin-screw extruder [38]. In a recent study, coriander press cake, a by-product of the oil extraction process, was used for the production of self-bonded boards with mechanical properties comparable to those of commercial wood-based panels such as particleboard and oriented-strand board (OSB) [20].

This study aimed to manufacture cohesive, renewable fiberboards from coriander straw, resulting in the valorization of this crop residue, while utilizing the binding capacity of coriander press cake through its addition as a natural binder to the board material prior to thermopressing. The effect of the addition of different amounts of press cake on the boards’ mechanical performance and water resistance were assessed. Alongside this, and with a view to improving the manufactured board properties, the effectiveness and feasibility of an innovative fiber pre-treatment process using twin-screw extrusion technology were evaluated and a comparison was made with a conventional grinding process using a hammer mill. The extrusion process with water injection presents a high pressure and shear hydro–thermo–mechanical pre-treatment, and different process conditions, representing different treatment severities, were tested through a variation of the liquid/solid (L/S) ratio of the material in the extruder. The implementation of a premixing stage was considered, which consisted of a twin-screw extruder passage of the board’s raw material, i.e., the straw and cake material, for intimate mixing and homogenization. Finally, the fiberboards were subjected to a heat post-treatment in order to enhance their dimensional stability.

## 2. Results and Discussion

### 2.1. Raw Material Characterization

The boards produced during this study incorporated two different raw materials. On the one hand, a press cake resulting from the mechanical pressing of coriander fruits for vegetable oil extraction was used after further deoiling by means of a solvent extraction. On the other hand, coriander straw was used as the main raw material, which is the lignocellulosic material consisting of the vegetative stalk parts of the coriander plant and represents the crop residue after the coriander fruits have been harvested.

Regarding the evolution of the oil content inside the press cake, the coriander fruits contained 28% vegetable oil on a dry matter basis. The thermomechanical oil pressing process resulted in a press cake with a residual oil content of 13%, while further deoiling of this cake through solvent extraction led to a deoiled cake material with a residual oil content of 0.9%. Deoiling of the press cake prior to thermopressing was carried out as previous studies have shown that the expression of residual oil in the cake during thermopressing may lead to the presence of defects inside the board, showing a detrimental effect on its mechanical performance [20,24]. Due to the extraction of vegetable oil from the material, the relative contents of the other compounds increased slightly. For example, the protein content of the coriander fruits was 14% of the fruit dry matter, while it was 25% for the press cake and 27% for the further deoiled press cake material.

The coriander straw and the deoiled press cake showed a moisture content of 8.9 ± 0.1% and 6.7 ± 0.2%, respectively. The cake consisted of a fine powder composed of almost spherical particles showing an average particle size of 54.6 μm, as determined through optical granulometry. The composition of the coriander straw and the deoiled press cake is presented in Table 1. Coriander straw is rich in cellulose (53%) and contains a relatively low amount of lignins (10%); most nonwoody, lignocellulosic materials show a cellulose and lignin content of 30–45% and 10–25%, respectively [39]. As such, coriander straw shows a similar composition to jute fiber and wheat straw [40,41]. Owing to its high cellulose content, which provides reinforcement for fiberboards, coriander straw shows potential as a raw material for the production of renewable panels with good mechanical performance. The self-bonding capacity of lignocellulosic material is often considered closely related to the presence of lignins. As such, the low lignin content of coriander straw may result in the production of fiberboards with weak internal bond strength. Here, the addition of a natural binder to the fiber material could provide a key solution as this has been shown to substantially improve a board’s performance characteristics, and most studies have focused on the addition of lignin as a natural bonding agent [28,42]. This study, however, aims to utilize the binding ability of the protein fraction of a deoiled press cake resulting from the extraction of coriander vegetable oil through twin-screw pressing to enhance the produced material properties. The cake is particularly rich in proteins (27%), which undergo plasticization upon thermopressing and thus exhibit a matrix effect inside the panels, embedding the fibers and resulting in cohesive, self-bonded materials [20,23]. Furthermore, this strategy provides an interesting means for the valorization of coriander press cake, which will become increasingly available in years to come owing to the recent industrial interest in coriander vegetable oil.

### 2.2. Fiber Refining Process

In order to enhance the mechanical performance and in particular the dimensional stability of the manufactured fiberboards, the coriander straw was subjected to a fiber refining treatment. For this, an innovative process using twin-screw extrusion technology was implemented, representing a high pressure and shear hydro–thermos–mechanical treatment that causes fibrillation of the fiber material. The straw feed rate was kept constant at 15 kg/h, while different flow rates of water were injected into the extruder barrel, corresponding to the L/S ratios of the material inside the extruder between 0.4 and 1. The extrusion temperature was maintained at 110 °C in order to ensure an intense steam-refining treatment of the fibers. For the purpose of comparison, a conventional grinding treatment of the coriander straw was also carried out using a hammer mill fitted with a 7.5 mm screen.

#### 2.2.1. Effect on Fiber Density and Morphological Characteristics

**Figure 1:**
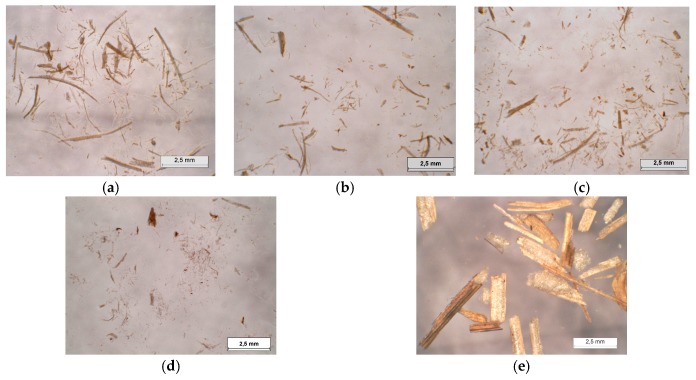
Macrographs of the coriander straw materials resulting from different refining processes. (**a**) Extrusion refining with a liquid/solid (L/S) ratio of 1.0; (**b**) Extrusion refining with a L/S ratio of 0.8; (**c**) Extrusion refining with a L/S ratio of 0.6; (**d**) Extrusion refining with a L/S ratio of 0.4; (**e**) Grinding process using a hammer mill.

The coriander straw material obtained after different refining processes can be observed in Figure 1, while their density and morphological characteristics are presented in Table 2. Firstly, when comparing the traditional grinding process with the extrusion process, it is clear that the extrusion pre-treatment results in a substantial improvement of the coriander fiber morphology, illustrated by a strong increase in the fiber aspect ratio from 4.5 to 23–27. It is well known that the fiber aspect ratio plays in important role in the mechanical properties of boards. The increased surface area of the defibrated material will result in enhanced fiber wetting and an increased bonding capacity. Therefore, it can be anticipated that the twin-screw extrusion pre-treatment will result in fiberboards with better overall performance. Besides this, a reduction in the density characteristics of the fiber material was observed with the extrusion treatment, which further demonstrates fiber fibrillation and destructurization, resulting in a more expanded, fluffy appearance of the straw. These structural differences are also illustrated in Figure 1. When comparing Figure 1a through Figure 1d, which represent the extrusion refined straw, with Figure 1e, which shows the milled straw, it is clear that extrusion results in significantly more advanced material defibration when compared with the grinding process using a hammer mill. Furthermore, the aspect ratios resulting from the morphological analyses in Table 2 are in agreement with the fiber aspects in Figure 1, even if the sample size represented by the macrographs is relatively small as compared to the morphological analysis (30,000 fibers per replication with four replications per sample). Secondly, when considering the varying L/S ratio applied during the extrusion process, representing different treatment severities, it can be seen from Table 2 that a reduction of the L/S ratio from 1.0 to 0.6 resulted in a slightly decreased fiber aspect ratio (from 26.5 to 25.2) and increased density (from 45 to 52 kg/m^3^). This can be attributed to the reduction in fiber length (from 547 to 515 μm) and the generation of fines due to the increased severity of the treatment with a lower L/S ratio. With a reduced amount of water, the material inside the extruder shows higher viscosity and thus undergoes stronger shear forces leading to the breakage of fibers [43]. This phenomenon is further illustrated by the increase in specific mechanical energy (SME) of the extruder, from 425 to 507 Wh/kg of straw (Table 3), and consequently, by the increased temperature of the material inside the barrel near the reverse screw elements (148 versus 151 °C), indicating increased self-heating upon high shearing. Furthermore, this tendency towards a decrease in the aspect ratio and an increase in the material density with lower L/S ratios was strongly intensified when an L/S ratio of 0.4 was applied during extrusion, indicating that high treatment severity results in important fiber breakage and a strong increase in material density and self-heating (up to 157 °C). The morphological results were confirmed by macrographs of the obtained extrudates. Indeed, when considering Figure 1a through 1d, representing extrusion L/S ratios from 1.0 to 0.4, respectively, increasingly shorter fibers can be observed, which is in agreement with the trend deducted from Table 2, where an increasing refining severity results in strong fiber cutting and the generation of fines. Similar effects have been observed by Xu et al. during the steam pre-treatment of kenaf core [8], where, on the one hand, an increased aspect ratio and decreased material bulk density were found following a mild pre-treatment, while on the other hand an increase of the treatment severity led to a reduction of the aspect ratio and an increase of the material density. 

#### 2.2.2. Economic Considerations

Table 3 presents the energy consumption of the extrusion pre-treatment and the fractions consumed by the extruder motor (SME), heating of the extruder (STE) and the cooling circuit (SCE), as well as the cost of the process and the total cost, which also includes the raw material cost. From this, it can be seen that the production of refined coriander straw material is slightly more expensive when a low L/S ratio is applied, which is mainly due to the increased power consumption of the extruder motor when processing material showing a higher viscosity, leading to stronger shear forces. This further leads to an increased specific cooling energy (SCE), which is necessary to counterbalance the phenomenon of the self-heating of the material due to high pressure and shearing in completely filled modules (i.e., the modules with restrictive screw elements such as mixing elements or reverse screws). A hypothesis for the elevated consumption of STE and SCE when applying an L/S ratio of 1.0, and thus for the concurrent lower energy consumption with an L/S ratio of 0.8, involves the relatively large amount of water present in the extruder with a L/S ratio of 1.0. This high water content results in an increased filling of the extruder barrel and may, on the one hand, lead to the increased self-heating of the material in the restrictive extruder zones, and thus to increased SCE consumption. On the other hand, the high water content and extruder filling may also lead to a less efficient heating/cooling process inside the extruder, consequently increasing the consumption of energy. Furthermore, the large amount of water is possibly not able to diffuse well into the material matrix, leading to the presence of extruder zones with higher or lower moisture contents, which may further induce the self-heating of the material and an inefficient heat transfer. For all L/S ratios, the mechanical energy consumption represents the most important part of the total energy consumption (63% to 69%). However, the production cost remains relatively low for all extrudates (maximum 0.065 €/kg of straw). The relatively low energetic cost of the extrusion process, combined with the low cost of coriander straw (0.09 €/kg), results in a relatively low total cost of extrusion refined coriander straw material of maximum 0.16 €/kg. For the fiber refining of the coriander straw material, the extruder feed rate was 15 kg/h while the screw speed was kept constant at 150 rpm. The maximal screw speed of the Clextral Evolum HT 53 twin-screw extruder being 800 rpm, an 80 kg/h maximal inlet flow rate could be accessible from such an extruder. Based on this 80 kg/h feed rate, a production time of 15 h/day and a depreciable life of 10 years for the twin-screw extruder, the global cost for the production of refined coriander straw (including the raw material cost and production cost, as well as the depreciation accounting of the extruder and staff expenses) would vary between 0.44 and 0.46 €/kg, depending on the L/S ratio (ranging between 1.0 to 0.4, respectively) applied during extrusion. Such a cost would remain competitive when compared to the cost of different commercial fibers such as hardwood and softwood fibers of comparable mean lengths (around 500 µm)—which are classically used for the mechanical reinforcement of thermoplastic matrices molded using extrusion or injection (around 0.50 €/kg)—or for bleached kraft pulp produced according to papermaking methods, i.e., digestion and defibration using a disc refiner (about 0.55 €/kg). When compared to traditional defibration methods such as a disk or conical refiner, twin-screw extrusion could result in important energy and water savings, while further reducing the amount of generated wastewater. This has been demonstrated for the fiber refining process for hemp fiber [44] and for rice straw [45]. Dean et al. reported an 87% reduction of the energy consumption, from 1.54 to 0.22 kWh/kg, for hemp fiber refining when using a twin-screw extruder, as compared to a double disk refiner [44]. A similar reduction in energy consumption was reported by Theng et al. [45] for rice straw, where the total specific energy consumption of twin-screw extrusion ranged from 0.67 to 0.95 kWh/kg, while this was between 6.18 and 8.52 kWh/kg for a digestion and rotating disc refining process. Moreover, twin-screw extrusion shows considerably higher comparable output, leading to increased productivity of the fiber refining process.

### 2.3. Thermopressing of Binderless Fiberboards

**Figure 2:**
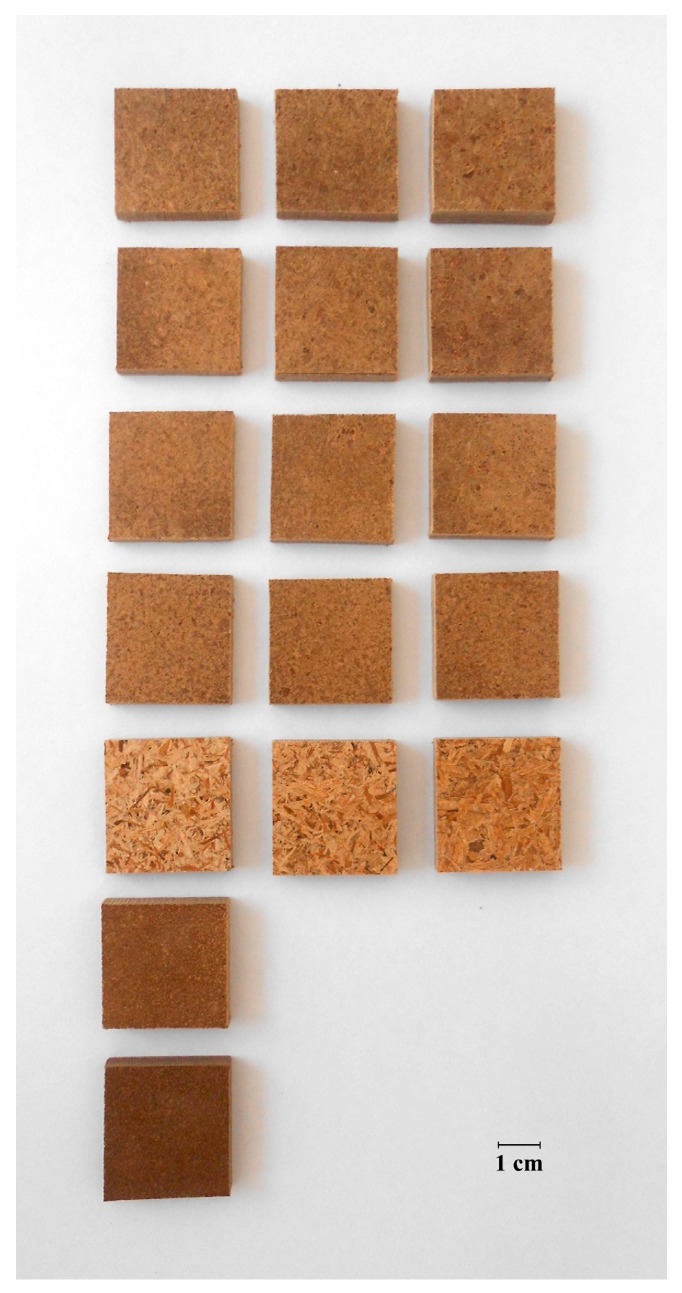
Test specimens of the binderless fiberboards produced during this study. Row 1–4, Fiberboards produced from extrusion refined straw with a L/S ratio of 1.0, 0.8, 0.6 and 0.4, respectively; Row 5, Fiberboards produced from milled straw; Columns 1–3, Fiberboards produced with a press cake content of 10%, 25% and 40%, respectively; Row 6, Fiberboard produced from premixed extrusion refined straw at L/S ratio 0.4 and 40% of press cake; Row 7, Fiberboard produced from premixed extrusion refined straw at L/S ratio 0.4 and 40% of press cake and subsequently heat treated at 200 °C.

The obtained extrudates and the ground coriander straw were subsequently used for the production of binderless fiberboards through thermopressing. In order to obtain cohesive panels with good performance, different amounts of deoiled coriander press cake (10, 25 and 40 wt % of the total raw material) were added to the raw material as a natural binder. The applied process conditions for thermopressing (21.6 MPa applied pressure, 205 °C mold temperature and 300 s molding time) resulted from a previous study in which the thermopressing process was optimized for the production of binderless boards from deoiled coriander press cake [20]. All raw materials were dried at 50 °C prior to thermopressing, resulting in moisture contents between 3.4% and 3.9%. Specimens of the produced fiberboards are presented in Figure 2. All manufactured fiberboards were cohesive, owing to the presence of lignins and particularly proteins as internal bonding agents, while the entanglement of lignocellulosic fibers from the straw material provided reinforcement. When comparing the fiberboards of Row 1 through 4, which were produced from extrusion refined straw with a decreasing L/S ratio, and Row 5, which was produced from milled straw, we can clearly see the important difference in fiber morphology for different refining processes. The fiberboards incorporating straw that was ground using a hammer mill show substantially coarser particles, which could also be deducted from Table 2, while the boards with extrusion-refined straw show a significantly more homogeneous aspect with an improved embedding of the fibers in the binder matrix. Increased extrusion severity, i.e., a lower L/S ratio, further enhanced the boards’ homogeneous aspect owing to the increased amount of fines. Increasing the press cake content inside the boards—represented by the different columns in Figure 2—resulted in slightly darker panels when milled straw was used, while it did not induce any visible differences for boards from extrusion refined straw. This further illustrates the proper coating of the defibrated straw material with the cake matrix and the absence of crude fibers on the panel surface in the case of extrusion refining. Finally, the fiberboards produced from premixed raw material (Row 6) and subsequently heat treated (Row 7) were significantly darker than non-premixed, non-post-treated boards. This darker aspect may, on the one hand, be attributed to the high process severity of multiple extrusion passages with concurrent self-heating, inducing darkening of the raw material. On the other hand, for the fiberboards that were thermally post-treated, darkening resulted from the partial degradation of the material and especially the formation of new bonds at 200 °C, a phenomenon that was also observed for the thermal treatment of materials from a sunflower press cake [21]. For all fiberboards, their performance characteristics, including density, flexural properties, impact properties, surface hardness and water sensitivity (i.e., both water absorption (WA) and thickness swelling (TS) after 24 h immersion in water), are presented in Table 4.

When considering the density of the manufactured fiberboards, values ranged between 1210 and 1220 kg/m^3^ for straw ground with a hammer mill, and between 1230 and 1330 kg/m^3^ for extrusion-refined straw without a premixing step or a post-treatment. The produced panels could thus be classified as high-density fiberboards (HDF) or hardboards (HB). It is important to note, however, that the obtained densities of the panels are significantly higher than the densities of most commercial boards and could result in industrial difficulties regarding their handling and transportation. The relatively high panel densities result from the absence of synthetic resins and is characteristic for binderless materials, which often show densities ranging between 800 and 1300 kg/m^3^ owing to the high pressure and temperature applied during thermopressing in order to mobilize the biopolymeric internal binders [3,30]. The higher densities obtained for extrusion-refined straw, as compared to ground straw, may be attributed to the decrease in particle size (Table 2), leading to a reduced resistance to compression. The increased amount of fines acts as a filler of voids inside the boards, improving the total contact between fibers and reducing board porosity. Further, the board density increases with a decreasing L/S ratio applied during extrusion, i.e., with increasing pre-treatment severity, and with increasing press cake addition. The latter results from the small, spherical particle size of the deoiled press cake (55 μm), filling the voids between the lignocellulosic fibers and showing high surface area, and the thermoplastic behavior of the protein fraction, providing good fiber wetting and a strong matrix effect, ultimately leading to cohesive fiberboards with strong internal bonding and reduced porosity. An increase in board density with increasing pre-treatment severity has been reported by many researchers and results from the increase in fines and the reduced average fiber length caused by the high pressure and shear conditions of high-severity straw pre-treatment [5,8,10,46].

**Figure 3:**
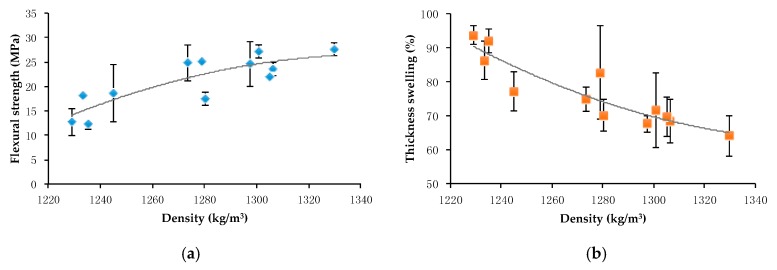
(**a**) Flexural strength and (**b**) thickness swelling as functions of panel density for the fiberboards produced from extrusion refined straw with varying L/S ratios and press cake addition.

**Figure 4:**
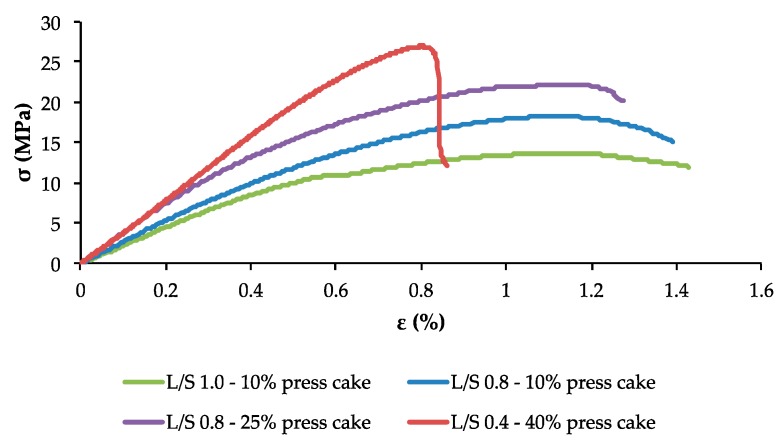
Flexural stress-strain curves determined through three-point bending tests of fiberboards produced with extrusion refined straw material using different liquid/solid (L/S) ratios and with different amounts of press cake.

It is well known that there is a good correlation between the density and the performance properties of materials [7,26,29,47]. Indeed, when looking at the flexural properties, the water resistance, and the surface hardness of the fiberboards produced from extrusion-refined straw, these characteristics steadily improve with increasing board density and thus with increasing treatment severity. This trend is further demonstrated in Figure 3, which presents the relation between the flexural strength (Figure 3a) or the thickness swelling (Figure 3b) and the density of the fiberboards. Further, it has been shown that the increased heat conductivity of materials with higher density leads to a reduced temperature gradient inside the board during thermopressing, resulting in better internal bonding and more homogeneous panels [11,26]. The flexural stress–strain curves for fiberboards produced from extrusion refined straw with different L/S ratios and with different press cake contents are shown in Figure 4. From this, it is clear that an increased extrusion pre-treatment severity (i.e., a lower L/S ratio) and an increased addition of press cake results in fiberboards with an enhanced mechanical resistance in terms of flexural properties. These improved properties are illustrated in Figure 4 through an increased stress at failure, representing the flexural strength, and an increased slope of the curve, implicating a greater elastic modulus. The panel showing the best mechanical properties, which include a flexural strength of 27.6 MPa, an elastic modulus of 4.5 GPa and a Shore D surface hardness of 79.7, was manufactured from coriander straw that was extrusion-refined with an L/S ratio of 0.4 and 40% of press cake addition, and showed a maximal density of 1330 kg/m^3^. The strongly defibrated straw material, showing an improved fiber morphology, certainly enhanced the bonding strength of the material and showed greater accessibility of the inner cell wall components such as lignins, further contributing to the self-bonding capacity. Besides this, an increased flow capacity of press cake material owing to its structural modifications—in particular defibration and protein denaturation—following an extrusion pre-treatment, has been demonstrated for sunflower cake [21]. The enhanced thermoplastic behavior of the material leads to improved fiber wetting and thus to boards with improved performance characteristics [48]. A variation of the severity of the extrusion refining process or the addition of press cake showed no significant influence on the impact strength of the fiberboards produced from extruded straw, which was always between 2.8 and 3.5 kJ/m^2^. However, this parameter was slightly higher for boards produced from conventionally milled straw (4.4–5.0 kJ/m^2^), owing to the increased average fiber length, providing toughness to the resulting panels.

When considering the mechanical properties of the fiberboards produced with straw that was ground using a hammer mill, the amount of press cake added shows a much less marked effect and does not significantly improve the boards’ mechanical performance when increased from 25 to 40%. Given the notably larger particle size of the straw material and thus the reduced surface area and available bonding sites, 25% of press cake may already be sufficient to generate the potential cross-linking and embed the fibers in a continuous matrix. Even though these fiberboards show a consistently lower density than the boards produced from extrusion-refined straw, their flexural properties are superior to those obtained with straw that was extruded with an L/S ratio of 1.0 when 10% or 25% of press cake was added, or with an L/S ratio of 0.8% or 0.6% and 10% of press cake. This may be due to the fact that the benefits of extrusion defibration were not fully exploited with only 10% or 25% of press cake, therefore being outweighed by the notably greater fiber length of the ground straw, imparting strength to the boards. However, for more severe extrusion refining of the straw, i.e., with an L/S ratio below 1, and with at least 25% of press cake, the mechanical performance of the boards was significantly improved as compared to conventionally ground straw, illustrating the effectiveness of the extrusion pre-treatment process.

In terms of water resistance, the manufactured fiberboards using extrusion-refined straw showed a thickness swelling of 94 to 64%, and 79 to 50% water absorption. The water sensitivity of materials is directly related to their porosity, and thus to their density [20,47]. Indeed, from Table 4, it is clear that fiberboards showing higher density also show lower water sensitivity, and the best dimensional stability (64% TS, 50% WA) was obtained with an extrusion pre-treatment at an L/S ratio of 0.4 and with 40% of press cake addition. The reduced water sensitivity results from the increased total fiber contact and reduced voids owing to the smaller particle sizes on the one hand, and the enhanced self-bonding of the material, on the other hand, with increasing straw pre-treatment or press cake addition. Besides this, fiber refining has been shown to result in the hydrolysis of hemicelluloses, which are highly hygroscopic compounds, thus resulting in important improvements in the dimensional stability of the produced fiberboards [5,28]. Further, when considering the fiberboards manufactured from milled straw, it is interesting to note that while their thickness swelling (86–72%) is situated within the range of the values obtained for the fiberboards from extrusion refined straw, their water absorption (140–110%) is much more important and significantly higher than the water absorption of extrusion refined boards (79–50%). This elevated water absorption may be explained through the lower density of the fiberboards produced from milled straw. Lower density values indicate higher material porosity, allowing stronger water uptake without necessarily concurrent thickness swelling.

### 2.4. Premixing Process of the Fiberboard Raw Material

The best fiberboard performance in terms of mechanical properties and water resistance was obtained with extrusion-refined coriander straw at an L/S ratio of 0.4 and with 40% of coriander deoiled press cake addition. Therefore, these production parameters were utilized in order to assess the implementation of a raw material premixing stage prior to thermopressing. Previously, the raw material, i.e., the coriander straw and press cake, was mixed manually before being thermopressed. However, with the aim of ensuring intimate mixing of both raw materials, a good distribution of the small, spherical press cake particles in the fibrous straw material, and therefore more homogeneous fiberboards with enhanced bonding and performance, a premixing operation could be included in the production process. Twin-screw extrusion is known to provide good product uniformity owing to its strong mixing capacity and high level of micromixing resulting from the interpenetration of the screws and the ability to introduce mixing blocks along the screw profile [49]. Hence, the raw material premixing stage was carried out in a twin-screw extruder equipped with two series of bilobe mixing blocks along the screw profile, while the refined straw material and the deoiled press cake were introduced at a 60/40 dry weight ratio at the feed inlet.

The performance characteristics of the fiberboard manufactured from the premixed raw material that was extrusion refined at an L/S ratio of 0.4 and with 40% of press cake, are presented in Table 4. When comparing these results to the properties of the corresponding fiberboard from non-premixed material (L/S ratio 0.4, 40% press cake), it is clear that the extrusion mixing process led to lower board density, decreased mechanical properties and improved dimensional stability. In order to gain a better understanding of this outcome, it is important to note that the additional passage through the twin-screw extruder, aiming at its intimate mixing, further imposes a high pressure and shear treatment on the raw material, thus substantially increasing the overall pre-treatment severity. Such high severity treatments inevitably lead to a strong reduction in fiber length and the generation of fines. While these fines improve the contact between fibers, leading to fiberboards with enhanced bonding strength and water resistance, the reduction of long fibers, which play the role of reinforcement in the boards through their entanglement, results in materials with decreased mechanical properties [5,10,46]. This is consistent with the obtained results, which include a significant decrease in flexural strength and elastic modulus from 27.6 to 21.5 MPa and from 4.5 to 3.5 GPa, respectively, and a notable decrease in thickness swelling from 64 to 49%. The shortening of the fibers is also illustrated by the further reduction in impact strength of the panels, from 3.3 to 2.5 kJ/m^2^. The increased water resistance of the fiberboards may also be attributed to the extended hydrolysis of hemicellulose compounds. Here, the significant degradation of the fiber material under the severe processing conditions should also be taken into account. Partial hydrolysis of lignins and cellulose has been reported for high severity treatments of kenaf core [8] and residual softwood [28], which resulted in reduced mechanical properties. Moreover, excessive degradation and denaturation reactions may be detrimental to the binder properties of the lignins and protein fraction, thus reducing the bonding strength of the resulting fiberboards [10,50]. The reduced bonding ability of the raw materials could then constitute a hypothesis for the reduced fiberboard density (from 1330 to 1240 kg/m^3^) observed after the inclusion of an extrusion premixing stage. Therefore, careful consideration and balancing of the fiber pre-treatment severity is often key to the production of boards with satisfactory performance.

### 2.5. Heat Post-Treatment of the Fiberboards

Thermal treatments have been applied to wood products in industrial practices in order to reduce their hygroscopicity and thus increase their dimensional stability. Recently, a post-treatment at 200 °C has also been shown effective in improving the water resistance of binderless materials from sunflower [21] and coriander [20] press cake material. Therefore, the fiberboard produced with premixed raw material was subjected to a heat treatment of 10 min at 200 °C in order to further reduce its water sensitivity. The results are presented in Table 4 and show that the applied post-treatment effectively decreased the thickness swelling by 50%, from 49 to 24%. Moreover, the board density decreased from 1240 to 1195 kg/m^3^, while the mechanical properties significantly improved, showing an increase in flexural strength of 35% (from 21.5 to 29.1 MPa) and in the elastic modulus of 9% (from 3.5 to 3.9 GPa). The increased water resistance of thermally treated boards results from crosslinking reactions of hemicelluloses, lignins and proteins induced by high temperatures increasing the mobility of polymeric chains and the reactivity of the ligneous and protein fractions [21,51]. Evaporation of water molecules during heat treatment also allowed the establishment of novel hydrogen bonds between the proteins and the cellulose hydroxyl groups [20]. This further contributed to the enhanced internal bonding of the boards, while reducing their hygroscopicity and thus their moisture uptake, which explains the decreased board density after conditioning. The replacement of water molecules, which exhibit a plasticizing effect, by polymeric interactions caused a rigidification of the fiberboards, illustrated by the increase in the elastic modulus. The same trends were observed during the thermal treatment of binderless materials from a sunflower cake, although a concurrent significant decrease in flexural strength was reported [21]. This loss in mechanical resistance has also been widely described for wood panels and is attributed to the degradation of lignocellulosic compounds under high temperature conditions [52]. However, as a hypothesis, the enhanced protein binding effect of the coriander cake material could outweigh the negative effect of degradation reactions and thus result in the net improvements in flexural strength observed during this study. 

### 2.6. Material Applications

The best performing binderless fiberboard obtained in this study was produced from premixed extrusion of refined (L/S ratio 0.4) straw and 40% of press cake, and subsequently heat treated at 200 °C. It showed a flexural strength of 29 MPa, an elastic modulus of 3.9 GPa and a thickness swelling of 24%. When compared to the binderless fiberboards obtained in other studies, the coriander fiberboards belong to the upper range of binderless panels in terms of mechanical performance and water resistance, as common figures reported for flexural strength and thickness swelling range between 13 and 35 MPa and 15% and 40%, respectively [3,30]. They are comparable to well performing binderless board produced from kenaf core [26] and oil palm trunk strands [53]. Besides this, the manufactured coriander binderless boards compare well with commercial wood-based materials such as oriented-strand board (OSB) and comply with international ISO requirements for general purpose medium density fiberboard (MDF), as well as furniture grade and load bearing MDF for use in dry conditions [54]. This allows their application for carcasses, furniture, cabinets, domestic flooring, shelving and general construction [55]. They further meet the requirements for furniture-type, load-bearing and heavy-duty load-bearing particleboards for use in dry conditions in terms of mechanical resistance, although the latter two classifications require further reduction of the thickness swelling to a maximum level of 22% and 16%, respectively [56]. The binderless boards do not meet the requirements for high-density fiberboards or hardboards (HDF), which include a flexural strength of at least 37 MPa.

## 3. Materials and Methods 

### 3.1. Materials

Coriander straw and fruits of French origin (GSN maintenaire variety) were supplied by GSN Semences (Le Houga, France). The press cake used in this study resulted from the vegetable oil extraction from coriander fruits using a Clextral (Clextral, Firminy, France) BC 21 twin-screw extruder with a screw configuration that was optimized in a previous study [38]. The operating conditions included a feed flow rate of 3.5 kg dry matter/h, a screw speed of 100 rpm and an extrusion temperature of 65 °C near the trituration zone and 74 °C near the pressing zone. Further deoiling of the press cake was carried out through a solvent extraction of 5 h with a 1 L Soxhlet apparatus and cyclohexane as the extracting solvent. Prior to the extrusion refining process, the coriander straw was crushed using an Electra Goulu N (Electra SAS, Poudenas, France) hammer mill fitted with a 12 mm screen. An Electra F3 (Electra SAS, Poudenas, France) hammer mill fitted with a 7.5 mm screen was applied for fiber refining. The composition of the coriander straw and deoiled press cake is presented in Table 1.

### 3.2. Twin-Screw Extrusion

**Figure 5:**
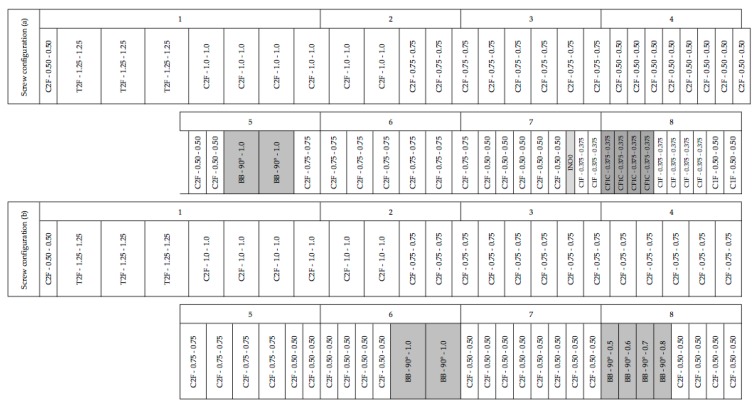
Screw configuration (**a**) for the coriander straw fiber refining process and screw configuration (**b**) for the raw material premixing operation, using a Clextral Evolum HT 53 twin-screw extruder. T2F, trapezoidal double-flight screws; C2F, conjugated double-flight screws; C1F, conjugated single-flight screws; CF1C, conjugated cut-flight, single-flight screws with left-handed pitch; BB bilobe paddles; INO0 linking element between double- and single-flight screws. The two numbers following the type of screw element indicate respectively the pitch and length of T2F, C2F, C1F and CF1C screws. The two numbers following the BB mixing blocks represent respectively the staggering angle and length. Shaded areas represent flow-restricting zones in the screw configuration.

A Clextral Evolum HT 53 (Clextral, Firminy, France) co-rotating and co-penetrating twin-screw extruder was used for the fiber refining of the coriander straw material and for the premixing of the refined straw and press cake material. The extruder barrel, with a length of 1.9 m, consisted of eight modules, each 4D in length (with D corresponding to the screw diameter, i.e., 53 mm), except for Module 1, which had an 8D length. Barrel modules 2 to 8 were temperature controlled. Feed material was introduced near the first module with a Coperion K-Tron SWB-300-N (Coperion GmbH, Stuttgart, Germany) gravimetric feeder. No outlet restriction was applied and the material exited the extruder barrel at atmospheric pressure. For the refining process, water was injected with different flow rates at the end of Module 3 using a piston pump DKM Super MD-PP-63 (Clextral, Firminy, France), while the straw feed rate was kept constant at 15 kg/h. For the premixing process, refined straw material and press cake were introduced at the extruder inlet with a 60/40 ratio based on the material dry weight and a feed rate of 54 kg/h was applied. The screw configurations that were applied for both operations are presented in Figure 5. The extruder screw speed was 150 rpm and the extrusion temperature was set at 110 °C for the refining operation and 80 °C for the premixing operation.

### 3.3. Thermopressing

A Pinette Emidecau Industries (Pinette Emidecau Industries, Chalon-sur-Saône, France) heated hydraulic press with a 400-ton capacity and a 150 mm × 150 mm aluminum mold was used for the thermopressing of the fiberboards. All raw materials were dried in a ventilated oven at 50 °C to a moisture content between 3% and 4% prior to thermopressing in order to avoid delamination of the panels. The material for the production of one panel consisted of 100 g of straw material and 10%, 25% or 40% of deoiled press cake based on the total raw material weight. The applied operating conditions were those optimized in a previous study [20] for the thermopressing of a deoiled coriander press cake and include an applied pressure of 21.6 MPa, a mold temperature of 205 °C and a molding time of 300 s. A degassing phase was added to the process in order to further reduce delamination, resulting in a three-step thermopressing process (i.e., compression, degassing, compression) with a second thermopressing cycle of 30 s. All manufactured fiberboards were conditioned in a climatic chamber (25 °C, 60% RH) for four weeks prior to their characterization.

### 3.4. Thermal Treatment

The thermal post-treatment of the fiberboards was conducted in a muffle furnace (Nabertherm GmbH, Lilienthal, Germany) and consisted of a temperature gradient of 25 to 200 °C at 7.5 °C/min and 10 min at 200 °C.

### 3.5. Analytical Methods

The moisture content, mineral content and residual oil content were determined according to ISO 665:2000, 749:1977 and 659:2009, respectively [57,58,59]. The cellulose, hemicellulose and lignin content were determined by the use of the acid detergent fiber-neutral detergent fiber (ADF-NDF) method of Van Soest and Wine [60,61]. The protein content was determined according to ISO 5983–1:2005 [62].

### 3.6. Morphological Analysis

Macrographs of extrusion refined and milled coriander straw materials were taken with a Leica/Wild M420 (Leica Microsystems, Wetzlar, Germany) ×8.75 macroscope, and the Archimed 4.0 (Archimed, Lille, France) software. Milled coriander straw was observed in a dry state. Extrusion refined fibers were first dispersed in an excess of water (i.e., 10 mg fibers for 20 mL water) for 90 min to break aggregates. The dispersed fibers were then positioned between two glass plates for analysis. The average particle size of the deoiled press cake and the morphological characteristics of the milled coriander straw fibers were determined through optical microscopy with a Nachet France (Nachet, Dijon, France) Z 45 P, ×15 binocular magnifier. Five different images were taken using the Archimed 4.0 (Lille, France) software and the measurements were made with the ImageJ (National Institutes of Health, Bethesda, MD, USA) software. The morphological characteristics of the coriander straw extrudates were determined using optical scanning of an aqueous fiber suspension in a flow cell. For this, a MorFi Compact Fiber & Shive Analyzer (Techpap, Gières, France) was used and results were processed with the MorFi v9.1.d (Techpap, Gières, France) software. The bulk and tapped densities of milled coriander straw fibers and of coriander straw extrudates were measured using a Granuloshop Densitap ETD-20 density tester (ACIL Sarl, Chatou, France).

### 3.7. Mechanical Properties

Flexural properties of the fiberboards were determined according to ISO 16978:2003 [63]. An Instron 33R 4204 (Instron, Norwood, MA, USA) universal testing system fitted with a 500 N load cell was used with the three point bending method and 30 mm wide testing samples. A grip separation of 80 mm and a test speed of 2 mm/min were applied. All determinations were carried out through four repetitions.

The impact properties of the unnotched test samples (60 mm × 15 mm) were determined according to ISO 179–1:2010 [64] and by the use of a Testwell Wolpert (Testwell, Gennevilliers, France) 0–40 daN cm Charpy testing apparatus. Analyses were carried out at 23 °C and the distance between the anvils was 30 mm. All determinations were made through eight repetitions.

The Shore D surface hardness of the fiberboards was determined according to ISO 868:2003 [65] and with a Bareiss (Bareiss Prüfgerätebau GmbH, Oberdischingen, Germany) durometer. All measurements were conducted through thirty-two repetitions.

### 3.8. Water Sensitivity

The water sensitivity of the fiberboards was evaluated through the determination of their thickness swelling (TS, %) and water absorption (WA, %) according to ISO 16983:2003 [66]. For this, 50 mm × 50 mm test specimens were immersed in distilled water at 20 °C for 24 h. All determinations were carried out through four repetitions.

### 3.9. Data Analysis

All data obtained from experimental determinations are expressed as the mean ± the standard deviation. Means were compared by the use of a one-way analysis of variance (ANOVA), using the GLM procedure of the SAS data analysis software (Cary, NC, USA). Comparison of individual means was performed using Duncan’s multiple range test at a 5% probability level.

## 4. Conclusions

In conclusion, the renewable binderless fiberboards from coriander straw and press cake show potential as viable alternatives to some current wood-based, resin-bonded products. These current commercial materials are not only becoming increasingly unsustainable due to the steady depletion of fossil and world forest resources, but also present a significant environmental and human health burden considering the use of toxic, hazardous synthetic resins during the production process and the emission of harmful volatile organic compounds such as formaldehyde. Therefore, the commercialization of coriander fiberboards could constitute a valuable solution owing to their bio-based nature, environmental friendliness and favorable cost–performance ratio. The valorization of readily available crop residues such as coriander straw, and process by-products such as press cakes, further adds to the sustainable nature of these materials and to the establishment of a true coriander biorefinery. Besides this, the use of a twin-screw extrusion process could present a cost-effective and environmentally friendly solution for fiber refining and could, as such, provide a valuable means for industrial process intensification.

Future research should focus on the further optimization of the binderless fiberboard production process and improvement of the boards’ dimensional stability. Dimensional stability is an important performance characteristic of fiberboards and presents one of the major challenges in the field of binderless materials. The hygroscopic nature of lignocellulosic plant material often leads to boards with unsatisfactory water resistance, which is detrimental for their industrial application potential. Therefore, research efforts should include the development of new methods to improve the dimensional stability of binderless materials. Alongside this, additional studies should be carried out regarding the optimization and feasibility of the raw material preparation process using twin-screw extrusion, which includes fiber refining and premixing. The extruder screw profile could be altered and/or the L/S ratio could be raised during fiber refining in order to reduce the overall pre-treatment severity. As a perspective, both processes could be integrated in a single extruder passage, which would not only allow a decrease in the production time, production cost and plant size, but would also bring about better preservation of the fiber structure due to the reduced residence time of the material inside the extruder, in turn resulting in enhanced material performance.

## Figures and Tables

**Table 1:** Chemical composition of coriander straw and deoiled press cake (% of dry matter).

Component	Coriander Straw	Coriander Deoiled Press Cake
Cellulose	52.5 ± 0.1	34.7 ± 0.4
Hemicelluloses	21.2 ± 0.5	36.9 ± 0.9
Lignins	9.8 ± 0.2	1.0 ± 0.1
Proteins	3.7 ± 0.1	26.7 ± 0.1
Lipids	0.8 ± 0.1	0.9 ± 0.3
Minerals	4.2 ± 0.2	6.7 ± 0.4
Hot-water extractives	10.4 ± 1.0	15.6 ± 1.0

**Table 2:** Density and morphological characteristics of refined coriander straw. L/S, liquid/solid.

Refining Process	Bulk Density (kg/m^3^)	Tapped Density (kg/m^3^)	Fiber Length (μm)	Fiber Diameter (μm)	Aspect Ratio (–)
Extrusion (L/S 1.0)	44.8 ± 0.5 ^a^	60.6 ± 0.3 ^a^	547 ± 21 ^a^	20.6 ± 0.3 ^a^	26.5 ± 1.0 ^a^
Extrusion (L/S 0.8)	46.8 ± 1.5 ^a,b^	63.6 ± 2.0 ^a^	534 ± 8 ^a,b^	20.4 ± 0.2 ^a^	26.2 ± 0.3 ^a^
Extrusion (L/S 0.6)	52.3 ± 0.1 ^b^	70.3 ± 0.1 ^b^	515 ± 12 ^b^	20.5 ± 0.1 ^a^	25.2 ± 0.6 ^b^
Extrusion (L/S 0.4)	81.8 ± 1.2 ^c^	110.0 ± 1.6 ^c^	485 ± 3 ^c^	21.2 ± 0.1 ^a^	22.9 ± 0.2 ^c^
Hammer mill	95.3 ± 4.2 ^d^	115.1 ± 2.7 ^d^	3541 ± 1357 ^d^	838 ± 335 ^b^	4.5 ± 1.7 ^d^

Means in the same row with the same superscript letter (a–d) are not significantly different at *p* < 0.05.

**Table 3:** Energy consumption and cost of the extrusion fiber refining process. L/S, liquid/solid; P, power consumption; SME, specific mechanical energy; STE, specific thermal energy; SCE, specific cooling energy; TSE, total specific energy.

L/S Ratio	P (W)	SME (Wh/kg)	STE (Wh/kg)	SCE (Wh/kg)	TSE (Wh/kg)	Production Cost ^1^ (€/kg)	Total Cost ^2^ (€/kg)
1.0	6585 ± 259 ^a^	425 ± 17 ^a^	134 ± 8 ^a^	119 ± 2 ^a^	677 ± 26 ^a^	0.054 ± 0.002 ^a^	0.144 ± 0.002 ^a^
0.8	7061 ± 269 ^b^	451 ± 17 ^b^	96 ± 16 ^b^	105 ± 2 ^b^	652 ± 35 ^b^	0.052 ± 0.003 ^b^	0.142 ± 0.003 ^b^
0.6	7849 ± 276 ^c^	507 ± 18 ^c^	95 ± 18 ^b^	151 ± 3 ^c^	753 ± 38 ^c^	0.060 ± 0.003 ^c^	0.150 ± 0.003 ^c^
0.4	8545 ± 363 ^d^	552 ± 24 ^d^	106 ± 18 ^c^	160 ± 5 ^d^	818 ± 46 ^d^	0.065 ± 0.004 ^d^	0.155 ± 0.004 ^d^

^1^ The production cost was calculated based on the cost of electricity in France (0.08 €/kWh); ^2^ The total cost represents the sum of the production cost and the raw material cost (0.09 €/kg of coriander straw). Means in the same row with the same superscript letter (a–d) are not significantly different at *p* < 0.05.

**Table 4:** Fiberboard characteristics produced from refined coriander straw and deoiled coriander press cake. WA, water absorption; TS, thickness swelling; PM, premixed raw material; PM-HT, premixed raw material and heat treated fiberboard.

Refining Process	Press Cake (%)	Density (kg/m^3^)	Flexural Strength (MPa)	Elastic Modulus (GPa)	Impact Resilience (kJ/m^2^)	Surface Hardness (–)	WA (%)	TS (%)
Extrusion (L/S 1.0)	10	1229 ± 27 ^a,b^	12.7 ± 2.8 ^a^	2.1 ± 0.2 ^a^	3.2 ± 0.3 ^a,b^	72.0 ± 2.2 ^a,b^	79 ± 1 ^a^	94 ± 3 ^a^
	25	1235 ± 34 ^a,b,c^	12.2 ± 1.0 ^a^	3.1 ± 0.1 ^b,c,d^	3.1 ± 0.2 ^a,b,c^	75.6 ± 2.4 ^c^	63 ± 1 ^b^	92 ± 4 ^a,b^
	40	1279 ± 48 ^c,d^	25.1 ± 0.8 ^b,c,d^	3.8 ± 0.2 ^e,f,g^	3.3 ± 0.4 ^a,b^	77.1 ± 3.0 ^d,e^	66 ± 4 ^b,c^	83 ± 14 ^b,c,d^
Extrusion (L/S 0.8)	10	1233 ± 42 ^a,b,c^	18.3 ± 0.4 ^e^	2.7 ± 0.2 ^a,b,c^	3.2 ± 0.3 ^a,b^	72.5 ± 2.5 ^a,f^	72 ± 2 ^d^	86 ± 6 ^a,b,c^
	25	1274 ± 23 ^a,c,d^	24.8 ± 3.7 ^b,c,d^	3.7 ± 0.1 ^d,e,f,g^	3.5 ± 0.7 ^a^	76.2 ± 2.1 ^c,d^	61 ± 5 ^b,e^	75 ± 4 ^d,e,f^
	40	1301 ± 17 ^d,e^	27.1 ± 1.3 ^b,c^	3.9 ± 0.2 ^f,g,h^	3.5 ± 0.3 ^a^	77.7 ± 2.7 ^e,g^	56 ± 1 ^e,f^	72 ± 11 ^e,f^
Extrusion (L/S 0.6)	10	1245 ± 57 ^a,b,c^	18.6 ± 5.9 ^e,f^	2.7 ± 0.1 ^a,b,c^	3.5 ± 0.5 ^a^	74.0 ± 2.3 ^h^	70 ± 7 ^c,d^	77 ± 6 ^c,d,e^
	25	1306 ± 15 ^d,e^	23.5 ± 1.4 ^c,d,f^	3.9 ± 0.1 ^f,g,h^	3.1 ± 0.4 ^a,b,c^	77.5 ± 1.8 ^e,g^	54 ± 1 ^f,g^	68 ± 6 ^e,f^
	40	1274 ± 26 ^d,e^	24.6 ± 4.6 ^b,c,d^	3.2 ± 0.4 ^b,c,d,e,f^	3.3 ± 0.4 ^a^	78.4 ± 1.8 ^g^	52 ± 3 ^f,g^	68 ± 2 ^e,f^
Extrusion (L/S 0.4)	10	1298 ± 4 ^c,d^	17.5 ± 1.4 ^e^	3.3 ± 0.3 ^b,c,d,e,f^	3.0 ± 0.4 ^a,b,c^	75.3 ± 2.2 ^c^	66 ± 4 ^b,c^	70 ± 5 ^e,f^
	25	1305 ± 35 ^d,e^	21.9 ± 0.5 ^d,e,f^	4.3 ± 0.3 ^g,h^	2.8 ± 0.4 ^b,c,d^	77.8 ± 2.1 ^e,g^	56 ± 2 ^e,f^	70 ± 6 ^e,f^
	40	1330 ± 15 ^e^	27.6 ± 1.3 ^b,c^	4.5 ± 0.4 ^h^	3.3 ± 0.3 ^a^	79.7 ± 2.1 ^i^	50 ± 3 ^g^	64 ± 6 ^f^
Hammer mill	10	1211 ± 9 ^b^	17.5 ± 1.4 ^e^	3.2 ± 0.3 ^b,c,d,e^	4.4 ± 0.4 ^e^	70.3 ± 2.7 ^j^	140 ± 2 ^h^	86 ± 2 ^g^
	25	1219 ± 14 ^b^	20.3 ± 2.0 ^d,e,f^	3.3 ± 0.4 ^c,d,e,f^	5.0 ± 0.3 ^f^	71.3 ± 2.5 ^b,j^	117 ± 3 ^a,d^	75 ± 1 ^h^
	40	1220 ± 7 ^b^	20.2 ± 2.0 ^d,e,f^	2.7 ± 0.3 ^a,b^	4.7 ± 0.5 ^e,f^	73.3 ± 2.5 ^f,h^	110 ± 7 ^d^	72 ± 1 ^h^
Extrusion (L/S 0.4) PM ^1^	40	1240 ± 38 ^a,b,c^	21.5 ± 2.1 ^d,e,f^	3.5 ± 0.9 ^d,e,f^	2.5 ± 0.3 ^d^	80.0 ± 1.7 ^i^	42 ± 1 ^i^	49 ± 1 ^i^
Extrusion (L/S 0.4) PM-HT ^2^	40	1195 ± 13 ^b^	29.1 ± 3.7 ^b^	3.9 ± 0.4 ^e,f,g,h^	2.7 ± 0.4 ^c,d^	81.1 ± 1.3 ^k^	24 ± 2 ^j^	24 ± 4 ^j^

^1^ Extrusion refining with subsequent premixing operation; ^2^ Extrusion refining with subsequent premixing operation and heat treatment of the fiberboard. Means in the same row with the same superscript letter (a–k) are not significantly different at *p* < 0.05.

## Abbreviations

HB	Hardboard
HDF	High-density fiberboard
L/S ratio	Liquid/solid ratio
MDF	Medium density fiberboard
OSB	Oriented strand board
P	Power consumption
PB	Particleboard
SCE	Specific cooling energy
SME	Specific mechanical energy
STE	Specific thermal energy
TS	Thickness swelling
TSE	Total specific energy
WA	Water absorption
